# Tensile creep of Norway spruce on the tissue scale

**DOI:** 10.1007/s11043-025-09772-1

**Published:** 2025-03-20

**Authors:** Alessia Ferrara, Falk K. Wittel

**Affiliations:** Institute for Building Materials, ETH Zurich, HIF E 28, Laura-Hezner-Weg 7, Zürich, 8093 Switzerland

**Keywords:** Wood tissues, Viscoelastic creep, Earlywood, Latewood, Moisture dependence

## Abstract

The rheological behavior of wood emerges from complex mechanical interactions occurring across multiple length scales. This behavior is characterized by directional dependence, as well as sensitivity to moisture content, loading time, and the degree of loading. This study focuses on the viscoelastic creep response of Norway spruce (*Picea abies*) tissues under different moisture levels and loading degrees. Using a custom-designed, fully automated test rack with moisture control, we investigate the uniaxial, moisture-dependent creep compliances across all feasible anatomical directions, as well as of isolated earlywood (EW) and latewood (LW) slices to understand their contribution to the cumulative behavior of the growth ring. The creep response is compared to the moisture dependence of the elastic compliance, revealing nontrivial scaling behavior as a function of moisture content. The results show significant directional dependencies and reveal the critical impact of moisture on deformation mechanisms. The transverse directions involve a complex interaction between bending, determining a more compliant and moisture-sensitive creep response, and cell wall stretching in the softest direction compared to loading in grain. These findings offer valuable insights into the moisture-dependent creep mechanisms of wood slices, highlighting the importance of exploring different orientations and tissues at various moisture content to fully understand the creep behavior at the bulk scale.

## Introduction

The complex cellular nature of wood plays a vital role for its mechanical behavior as it involves diverse morphology, material properties, and interactions across multiple intrinsic scales. Each growth ring of softwood is formed by a bundle of tracheids spanning from EW to LW and consists of multilayered cell walls with variable thickness, microfibril angle (MFA), and proportions of the basic chemical components (cellulose, hemicellulose, and lignin) with distinct mechanical properties. The hierarchical structure poses significant challenges (Fratzl and Weinkamer 2007) when investigating the mechanical behavior of wood at submacroscopic scales. Despite decades of experimental and numerical studies, a comprehensive understanding of wood’s viscoelastic behavior has yet to be achieved on the tissue scale. In the long run, this knowledge is essential for improving the reliability of performance predictions, especially in innovative applications for modern constructions (Alrubaie et al. 2020; Brandner et al. 2016, 2018; Grönquist et al. 2019; Jeleč et al. 2018; Nguyen et al. 2019).

An extensive body of research has been conducted on bulk wood across various species, investigating the creep behavior under tension, compression, bending, and shear in multiple anatomical directions (Niemz et al. 2023). In general, studies consistently demonstrate that viscoelastic creep is strongly influenced by loading direction (Ando et al. 2023; Cariou 1987; Hayashi et al. 1993; Hoyle et al. 1985; Liu 1993; Ożyhar et al. 2013; Schniewind and Barrett 1972; Shimazaki and Ando 2024), magnitude of the applied load (Jiang et al. 2016), and environmental conditions (Aicher et al. 1998; Armstrong 1962; Bach 1966; Jong and Clancy 2004; Schniewind 1968). The observed viscoelastic response is typically characterized using combinations of rheological models like springs for the elastic behavior and dashpots to represent the viscous one (Navi and Stanzl-Tschegg 2009; Tong et al. 2020). Unfortunately, incomplete datasets complicate the holistic calibration of these models. Scaling or combination of available data from various studies, anatomical directions, or across species (Fortino et al. 2009; Hanhijärvi and Mackenzie-Helnwein 2003; Hassani et al. 2015) is consequently an emerging source of discrepancies between predicted and observed behavior. On the macroscale, there is a recently published comprehensive dataset on Norway Spruce (*Picea abies*) (Maas and Wittel 2025). Studies on the micro- and ultrascale explored the viscoelastic properties of wood fibers and cell walls, focusing on their varying chemical compositions and MFA (Cai et al. 2022; Dong et al. 2010; Wang et al. 2021), as well as the influence of humidity (Meng et al. 2015). Neither macro- nor microscale behavior can be directly related to the one of the tissue scale where experimental studies are sparse, focusing mainly on creep parallel to grain (Eriksson and Norén 1965; Robson 1989). Typically, the influence of the MFA (El-Osta and Wellwood 1972; Kojima and Yamamoto 2004; Roszyk et al. 2010, 2011) is addressed, but rarely the role of moisture (Engelund and Salmén 2012; Pittet 1996) or long time spans. To the authors’ knowledge, no comprehensive study has been conducted on the creep behavior of wood tissues under varying humidity levels, loads, and directions.

The cellular structure accounts for the wide variability in macroscopic properties, bridging the bulk wood with the chemical organization within the tracheids. Therefore, a thorough characterization of the different wood tissues is essential to fully understand the behavior at the bulk scale. This study focuses exclusively on the viscoelastic creep behavior on the tissue scale for various tissue types under varying relative humidity (RH) and loading degrees (LD) across all feasible anatomical directions. We explore longitudinal (L), radial (R), and tangential (T), as well as earlywood (EW) and latewood (LW) slices, to understand their contribution to the cumulative viscoelastic behavior of bulk wood. In general, the mechanical impact of ray tissue is negligible for Norway spruce (Burgert et al. 2001). However, on the tissue scale, it should be considered to interpret the observed behavior.

A fully automated test rack with moisture control was constructed for the simultaneous testing of multiple samples over long time spans to explore the combinatory variety of tissues under various orientations, RH levels, and LDs. The resulting creep compliances $J_{c}$ are characterized by Kelvin–Voigt (KV) series-based models. This way, one can compare uniaxial moisture-dependent components of the viscoelastic creep compliance tensor $J_{c,ii}$ for a single tree. By analyzing the relationships between the individual components as well as their dependence on moisture content $mc$ ($J_{ii}(mc)$), we propose a directional classification that could potentially reduce the experimental effort.

## Materials and methods

In this section, the preparation of samples for tensile creep testing across various anatomical directions is first discussed in detail (Sect. 2.1). Then, the automated, climatized creep rack is explained along with the control and data acquisition capabilities in Sect. 2.2. The section concludes with a comprehensive explanation of the data processing and analysis procedures, emphasizing the techniques used to ensure the accuracy and reliability of the results in Sect. 2.3.

### Material selection and sample preparation

All samples were extracted from a log of a 118-year-old Norway spruce tree harvested from the Bannwald in Alpthal (Switzerland) at an altitude of 1093 m. The log was cut in a green state about 1 m above the ground and segmented to prevent cracking during drying at controlled conditions of 65%RH/20 °C. Once equilibrated, differently oriented blocks of size (20 × 15 × 50) mm^3^ were extracted for further sample preparation. To minimize microdamages, all blocks were immersed in demineralized water for three days prior to slicing. All tissue slices were then prepared in a saturated state using a rotational microtome (Leica RM2255). After slicing, the samples were stored in demineralized water for two more days to reduce potential residual stress. Subsequently, the samples were dried and stored under environmental conditions. Note that in a preceding study, the same procedure was followed to determine the skleronomous, moisture-dependent elastic compliances $C_{0}^{-1}$ and strengths $\sigma ^{f}_{ii}$ (Ferrara and Wittel 2024). In contrast, this study focuses on the rheonomous behavior at the tissue scale.

**Fig. 1:**
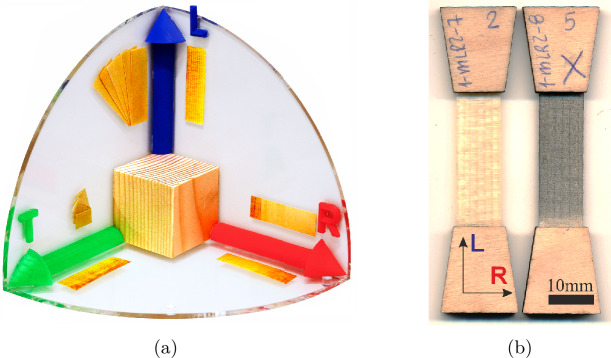
Spruce tissues for all combinations of directions {*LR, RL, RT, TR, LT, TL*} (a), and exemplary *LR* samples prepared with glued-on load application pieces from plywood, and speckled for DIC measurements via an airbrush (b)

Tissues for all combinations of directions were obtained, i.e., {*LR, RL, RT, TR, LT, TL*} shown in Fig. 1a. The first letter defines the loading direction, and the second indicates the transverse width. Samples {*LR, RL, RT, TR*} are characterized by alternating bands of EW and LW, while for {*LT, TL*}, different EW and LW tissues were isolated, namely {*LT-EW, LT-LW, TL-EW, TL-LW*}. Considering that LW occupies only a thin portion of the growth ring and the ring itself is not perfectly straight, isolating pure LW by cutting samples near the interface is highly challenging. Since tissue classification relies on visual inspection during the cutting process, the tested LW may contain small traces of transition wood (TW) from the same growth ring or EW from the subsequent one. However, the presence of small traces of other tissues has a minor effect on the resulting mechanical properties and can be neglected. All samples measured about 50 mm in length and 10 mm in width (except *LT-LW* with 5 mm width). The thickness varied by tissue type: 0.3 mm for {*LR, LT-EW, TL-EW*}; 0.15 mm for {*TL-LW, LT-LW*}; 0.35 mm for {*RL, RT, TR*} to meet the loading ranges of the creep rack. However, for {*RL, RT, TR, TL*}, preliminary tests revealed that at least double the thickness was necessary (see Sect. 2.2). Consequently, two slices were glued together at their ends to form double-thickness samples. Trapezoidal load application pieces from 2 mm thick plywood were glued to each sample’s ends to ensure a smooth load introduction (Fig. 1b) and sample alignment. This reduced the effective measurement length to 30 mm. For {*TL-EW, TL-LW*}, the growth ring curvature limited the maximum length to 15 mm. The sample length was increased using glued-on strips, which reduced the effective length for strain measurements of those samples to 10 mm. Finally, since the strain measurement was accomplished via Digital Image Correlation (DIC) (see Sect. 2.3), all samples were speckled with fine black and white acrylic paint dots from both sides with an airbrush to obtain a characteristic pattern (Fig. 1b). An overview of the 253 samples that entered this study is provided in Table 1 with at least $n=5$ experiments for all relevant situations. Note that such experiments, where early failure occurred, pre-damage from sample preparation or clamping was evidenced, or DIC measurements were not trustworthy, did not enter the study.

**Table 1:** Sample sizes for the respective cases, grouped by moisture content $mc$ and loading degree LD

Loading direction	Transverse direction	Tissue type	LD [%]	30			50		
			*mc* [-]	0.07	0.12	0.2	0.07	0.12	0.2
*R*	*L*			5	5	5	5	6	6
*R*	*T*			5	6	5	6	5	7
*L*	*R*			6	6	5	6	6	5
*L*	*T*	*EW*		5	6	5	5	6	6
		*LW*		7	6	7	6	6	6
*T*	*R*			5	6	6	5	6	5
*T*	*L*	*EW*		6	5	5	5	5	5
		*LW*		3	3	3	5	3	1

### Automated creep testing

**Fig. 2:**
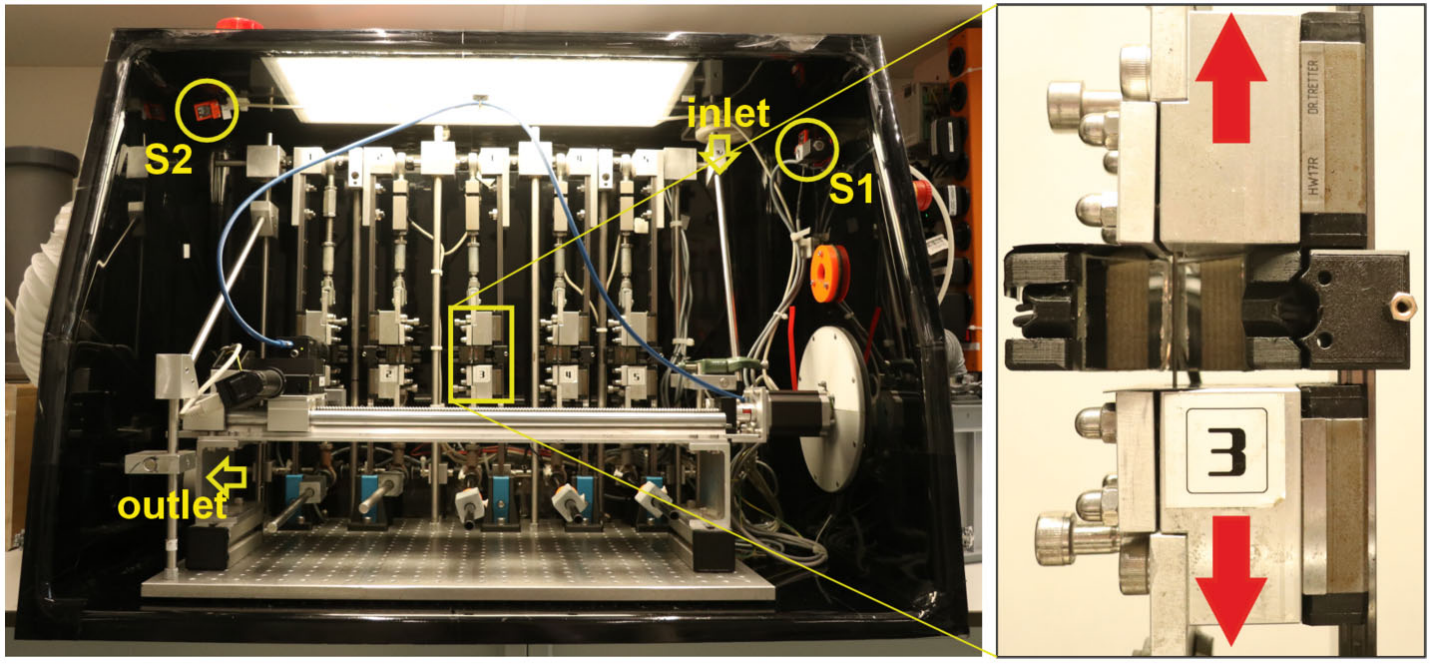
Custom-designed test rack for tensile creep testing on thin tissue samples, equipped with five sample holders (inset) to allow simultaneous testing of multiple samples. The humidity sensors (S1, S2), along with the inlet and outlet of the ventilation system, are marked to highlight their positions

Creep testing for such a large amount of samples, under various orientations, loading degrees, or humidity, requires a high degree of automation. These challenges were met by designing a 5-axis creep test rack, small enough to fit in a glove box (see Fig. 2). Four of the axes are equipped with load cells (5 kN ALMEMO 710). The sample holders are mounted on carriages on linear guide rails with low translational resistance to ensure purely uniaxial motion. The load is applied via horizontal loading lever bars attached to the bottom linear guide carriage, where static metal weights can be positioned along the bar to realize loads from 5 to 700 N. Each sample holder has a dual-mirror system (Fig. 2), acquiring images of both sample surfaces. This setup enhances the accuracy of the DIC analysis and eliminates errors from potential out-of-plane movement. The camera (Grasshopper 3 GS3-U3-28S4C) is mounted on a two-axis motion system, allowing it to sequentially capture both sides of the five samples (axis 1) and to individually set the distance to the focal plane since the focus of the camera lens is fixed (axis 2). The relative humidity inside the glove box can be controlled in a range from 20 to 95%. A Raspberry Pi 3B+ runs a Python-based control system that reads data from two moisture sensors (BME680, $\pm 3\%$ RH accuracy) placed on opposite sides of the glove box, marked in Fig. 2. One sensor is near the inlet, and the other is positioned in the farthest corner from the in- and outlet to measure humidity at different locations. Based on their mean value, the system regulates humidity by opening a valve to introduce dry air (at 10% RH) when RH exceeds the target or activating a water nebulizer if RH falls below it. A ventilation system ensures uniform climatic conditions inside the box. The master control for the camera positioning, image acquisition, data management, and RH target values for the Raspberry Pi are realized via LabVIEW scripts.

The samples were tested at three levels of constant RH: 30, 65, and 90%, corresponding to average $mc$ of 0.07, 0.12 and 0.2, for seven, five and three days, respectively. Note that the dynamic vapor sorption (DVS) test results for early-, transition-, and latewood are provided in Appendix A. Moreover, two LDs of approximately 30 and 50% of the tensile strength $\sigma ^{f}_{ii}$ were studied for each type of sample. The reference values of $\sigma ^{f}_{ii}$ corresponding to 30, 65, and 90%RH were previously determined in Ferrara and Wittel (2024) and are summarized for completeness in Table B1.

### Data processing and analysis

The base of the evaluation consists of two series of time-indexed images of the speckled surfaces per sample. Note that all images are corrected for lens distortion before processing. The two surface strain fields are calculated using DIC of reference and respective current configuration images at time $t$ with the Ncorr package for Matlab (Blaber et al. 2015), with estimated strain accuracy of about $\pm 3\cdot 10^{-5}$. Finally, only one scalar strain value for the tensile strain in the loading direction is needed. To avoid edge effects, it is taken as the average strain component of approximately the inner 30% to 50% of the surfaces. Errors due to eventual sample motion with respect to the focal plane are eliminated by averaging the scalar strain values of both surfaces. The average tensile strain $\varepsilon (t)$ is then synchronized with the respective load and RH data. Note that transverse strains were evaluated but finally omitted from this study since they are relatively small with respect to the measurement accuracy of the procedure. With the creep stress $\sigma _{0}$, calculated from the initial cross sections and the applied load, the compliance $C^{-1}(t)$ is calculated as $C^{-1}(t)=\varepsilon (t)/\sigma _{0}$.

**Fig. 3:**
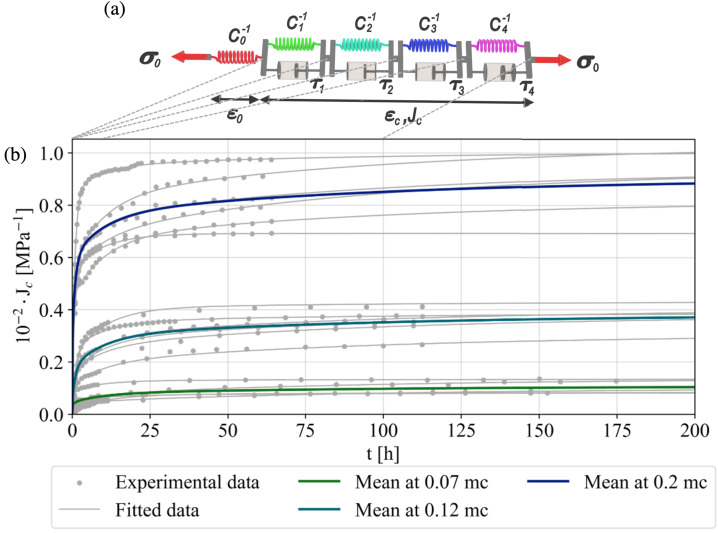
Scheme of the Kelvin–Voigt series model (a) with $N=4$ elements, each consisting of a spring and a dashpot in parallel, used to fit the experimental creep compliances $J_{c}$ here exemplarily for RL samples at different moisture content $mc$ and 50% loading degree (b)

Since $C^{-1}$ is the total compliance, it is composed of the elastic $C_{0}^{-1}$ and creep compliance $J_{c}(t)$, namely $C^{-1}(t)=C_{0}^{-1}+J_{c}(t)$. In this work, $J_{c}$ was modeled using a series of KV elements (Fig. 3a) within the framework of linear viscoelasticity (Hayashi et al. 1993; Navi and Stanzl-Tschegg 2009). The KV series-based model is particularly suitable for representing the creep behavior, as widely recognized in the literature (Gutierrez-Lemini 2014; Hajikarimi and Moghadas Nejad 2021). The KV elements, which are serially superimposed with the instantaneous elastic response, exhibit varying time delays, enabling the model to capture different time spans of strain history under constant stress. This setup also allows for a straightforward extension with other moisture-dependent rheological properties (Hassani et al. 2015). The mathematical formulation of this model is expressed by

1 $$ J_{c}(t) = \sum _{i=1}^{N}C_{i}^{-1}(1-e^{-t/\tau _{i}}), $$

where $N$ denotes the number of KV elements, $C_{i}^{-1}$ is the element compliance, and $\tau _{i}$ denotes the relaxation time of KV element $i$. We consistently use $t$ and $\tau $ in hours, and all compliances in MPa^−1^. Note that unloaded samples can exhibit warping and misalignment inside sample holders and are, therefore, not a good choice as reference images. Since elastic compliances are not needed for a creep study, we circumvent this problem by taking the first image after the load application as the reference image for the DIC. The unknown model parameters, $C_{i}^{-1}$ and $\tau _{i}$, were determined by least squares fits to the experimental data (see Equation (1)). While increasing the number of parameters allows for a better fit to the experimental data, it also necessitates a larger number of experiments for identification and complicates the interpretation of the physical meaning of the rheological elements (Eitelberger et al. 2012). To determine the optimal number of KV elements, the Fisher test (F-test) was performed on each dataset with a significance level of $\alpha =0.05$. The F-test is a standard statistical method used to compare nested models and to assess whether adding an additional KV-element improves the model fit (Hajikarimi et al. 2018). As $N$ resulted from 3 to 4 for most tests, $N=4$ was taken for this work. To ensure consistency in the fitting process and to reduce the number of unknown parameters, the values of $\tau _{i}$ were fixed *a priori* at [0.1, 1, 10, and 100] hours for all experiments. These values were selected to be logarithmically spaced, covering a timescale that encompasses the duration of all experiments.

Different approaches were compared to average up to 7 experiments for a single case. The most robust, finally chosen one, first fits the 4-element KV-series to each experiment to be able to calculate the creep response at any given time inside the test time interval (lines with markers in Fig. 3). In the consecutive step, at discrete time intervals, average creep responses are calculated from the individual KV-series, and a mean 4-element KV-series is determined for the case (colored lines in Fig. 3). Those are further processed to predict $\overline{J_{c}}(t)$, which describes the average viscoelastic behavior of spruce tissues for each situation investigated and is further used in the analysis.

## Results

The study provides the experimental basis for a thorough understanding of how moisture and applied load influence the creep compliance $J_{c}$ of different spruce tissue. First, we provide an overview of our findings by exploring the dependence of creep strain $\varepsilon _{c}$ and $J_{c}$ on $mc$ and applied load. Next, we study how $J_{c}$ scales with the elastic compliances $C_{0}^{-1}$ reported in our previous work (Ferrara and Wittel 2024), providing a comprehensive picture of the distinct impact of $mc$ on the material’s elastic and creep behavior with respect to loading orientations.

### Moisture- and load-dependence of viscoelastic creep

**Fig. 4:**
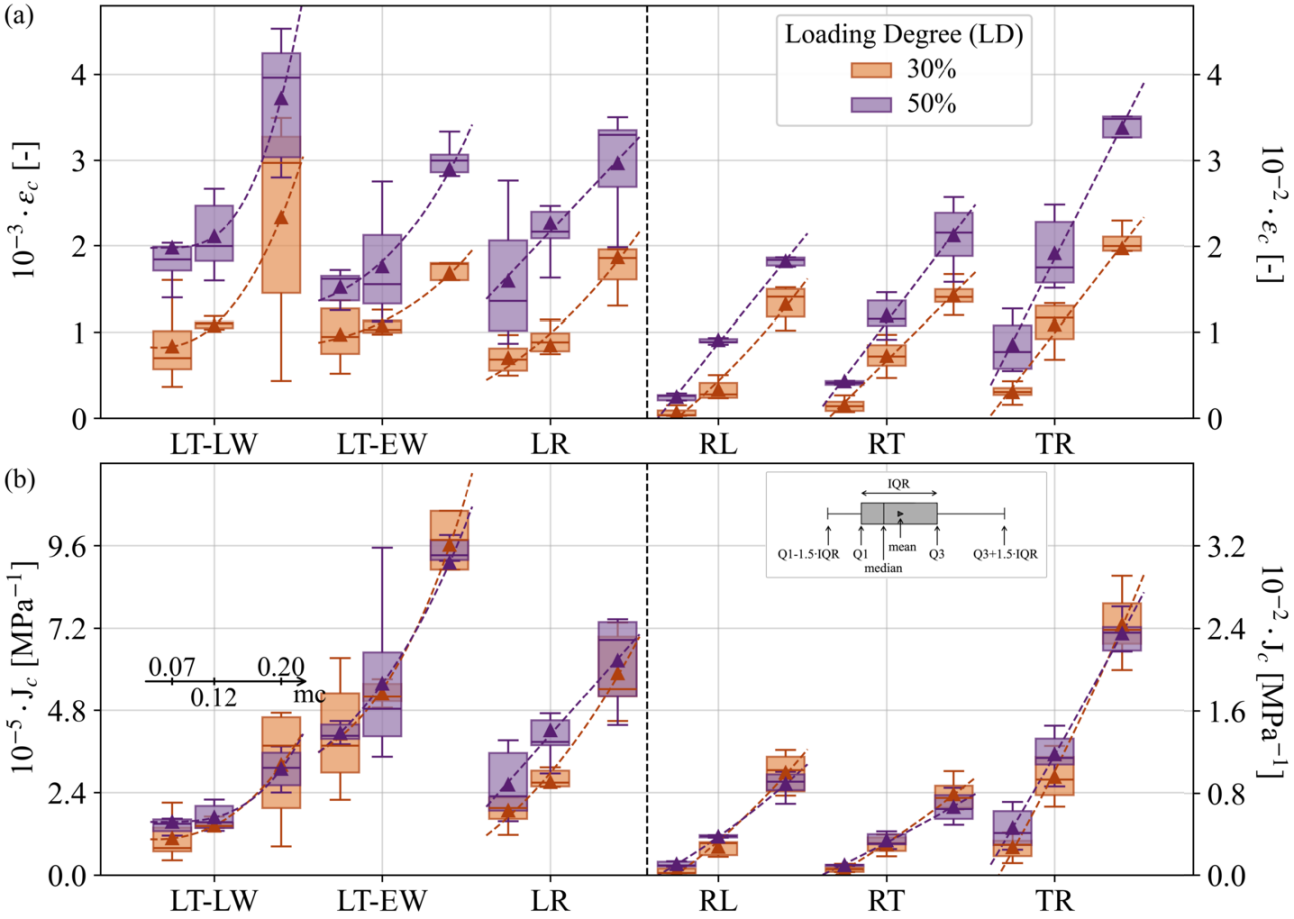
Predicted values of creep strain $\epsilon _{c}(t=150)$ (a) and creep compliance $J_{c}(t=150)$ (b) in MPa^−1^ for different loading degrees LD (30 and 50%) at three moisture content $mc$ values 0.07, 0.12, and 0.2, corresponding to 30, 65, and 90% relative humidity, respectively. The values are calculated at $t=150~\text{h}$ of creep

To prepare data for this comparative study, all cases are evaluated with the procedure described in Sect. 2.3. Since creep strains and compliances are functions of time, a fixed point in time at $t=150~\text{h}$ is chosen for comparison. The data is aggregated in Fig. 4a as boxplots of creep strains $\varepsilon _{c}(150)$ for each sample type, grouped by loading degree (LD) and $mc$. Using the average strain values for each case, a trend line shows a parabolic dependence of the creep strain on $mc$. A clear trend is visible as higher applied loads increase creep deformation. This effect is even amplified at high moisture levels (Roszyk et al. 2011), as evidenced by the diverging curves.

Dividing the creep strain by the applied load results in the creep compliance $J_{c}$. In linear viscoelasticity, identical values of $J_{c}$ are calculated for different loading degrees. In Fig. 4b one can observe how the compliances for the two loading cases collapse. This observation confirms, consistently with literature (Hayashi et al. 1993), that the viscoelastic response remains within the linear domain at least up to 50% LD. Notably, the trends for both strain and compliance reflect a non-linear response to increasing moisture, which is more pronounced in the L-direction compared to the transverse directions. This points to directional dependencies of the underlying deformation mechanisms.

At first sight, early- and latewood creep strains do not seem to differ too much (see Fig. 4a). However, one should remember that the load on EW is smaller due to identical LD but lower strength of EW as well. The accurate picture is given by the compliances, where it is evident that EW creeps stronger than LW, with *LR* being in between, as both tissue types are present (see Fig. 4b). Unfortunately, the results for the *TL-LW* and *TL-EW* samples had to be excluded due to unacceptable data scatter, likely caused by imprecise load control in the test rack and challenges in preventing glue penetration or ensuring flatness of the slices during the gluing process.

### Moisture impact on creep *vs* elastic compliances

**Fig. 5:**
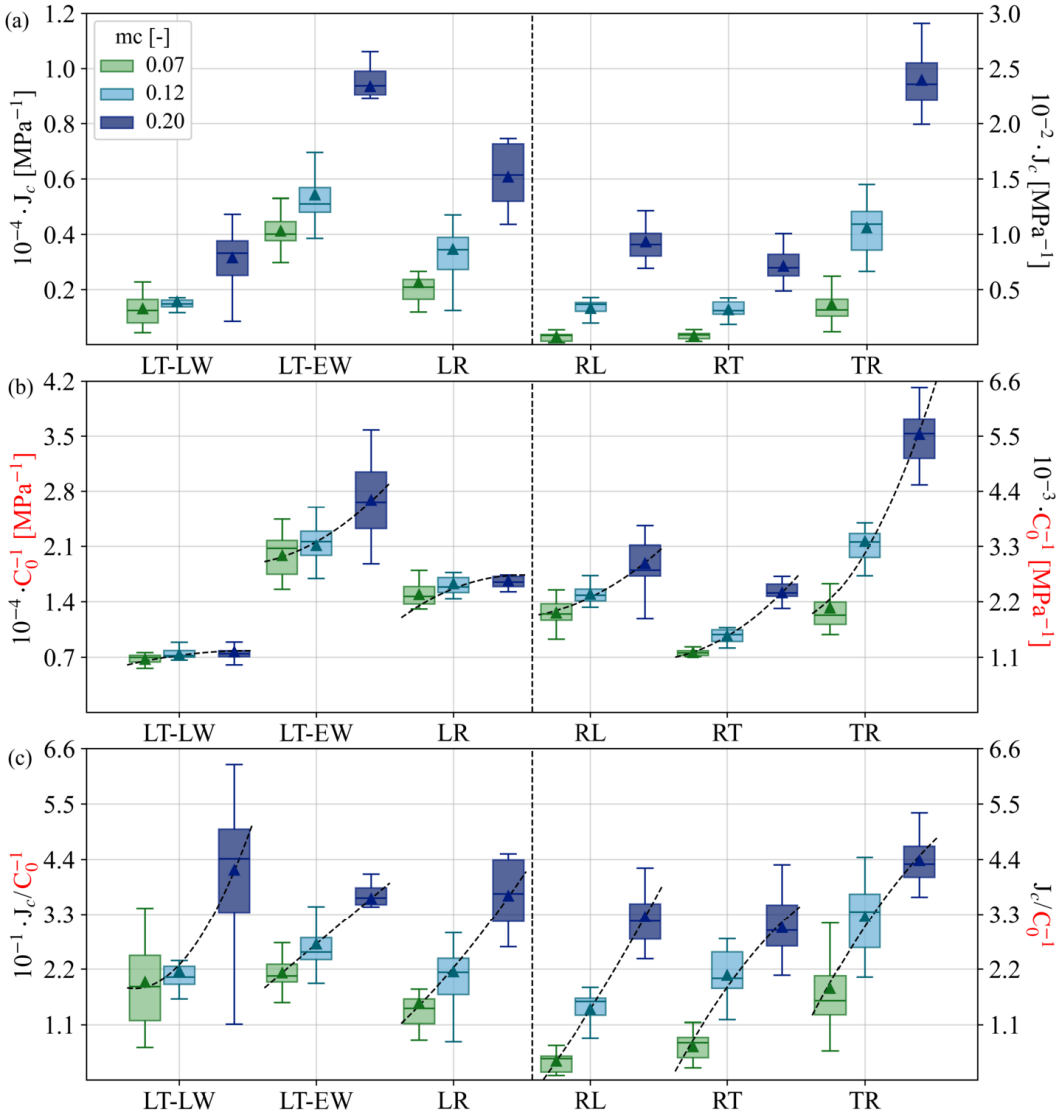
(a) Predicted results of creep compliance $J_{c}(t=150)$ in MPa^−1^ combining loading degrees LD (30% + 50%). (b) Experimental results of linear elastic compliance $C_{0}^{-1}$ in MPa^−1^ (Ferrara and Wittel 2024). (c) Normalized creep compliance to linear elastic compliance $J_{c}(t=150)/C_{0}^{-1}$. Values are sorted by moisture content $mc$ at 0.07, 0.12, and 0.2, corresponding to 30, 65, and 90% relative humidity, respectively. Creep values are calculated at $t=150~\text{h}$

Since the collapse of creep data for different loading degrees was just demonstrated (Fig. 4b), one can make use of it and cumulate the different LDs (30% + 50%). This gives a clearer picture of the relationships between the directions, as the amount of data is more than doubled (see Fig. 5a). Consistent with previous studies (Hayashi et al. 1993; Ożyhar et al. 2013; Taniguchi et al. 2010), $J_{c}$ in the R-direction falls between that of the L- and T-directions, reflecting a pattern similar to elastic deformation behavior (Ferrara and Wittel 2024). In the L-direction, EW still shows higher $J_{c}$ compared to LW, with combined EW/LW samples falling in between. In the R-direction, a comparable trend was expected for *RT* and *RL*, given their shared loading direction. However, *RL* tends to exhibit greater compliance under high moisture conditions.

It is evident that the creep compliance $J_{c}$ increases with moisture, but this increase varies significantly across the anatomical directions, particularly in R and T. However, it is known that the elastic compliances $C_{0}^{-1}$ also increase with moisture (see Fig. 5b made with data from Ferrara and Wittel (2024)). If both compliances were equally affected by moisture, their ratio $J_{c}/C_{0}^{-1}$ would be independent of the moisture. Note that this relates to the strain ratio $\varepsilon _{c}/\varepsilon _{0}+1$. In Fig. 5c, one can, however, observe that the increasing trend with moisture remains. This points to the more pronounced sensitivity of creep $J_{c}$ to moisture compared to elasticity. Even though we only show numbers for $t=150~\text{h}$, the trend is as follows: the $J_{c}$ in L-direction remains consistently lower than $C_{0}^{-1}$ for all moisture levels. In contrast, in the T-direction, $J_{c}$ consistently exceeds $C_{0}^{-1}$. Meanwhile, in the R-direction, $J_{c}$ surpasses $C_{0}^{-1}$ as $mc$ increases. Interestingly, for EW, LW, and their combination, the ratios remain comparable up to 0.12 $mc$, namely within the chemisorption-dominated range. The ratio increases sharply at higher moisture levels, where physisorption and capillary sorption prevail, and liquid water is present within micropores. In particular, for LW, one observes a disproportionate acceleration of $J_{c}$ compared to $C_{0}^{-1}$. In contrast, $J_{c}/C_{0}^{-1}$ for EW increases in a near-linear manner. While T is more affected by creep than R, both directions show a pronounced increase in the chemisorption range (0.07 to 0.12 $mc$). At higher moisture levels, $J_{c}$ still increases faster than $C_{0}^{-1}$, but one can guess convergence to a constant ratio as fiber saturation is approached. Note that one can easily calculate the average creep response for arbitrary times using the KV-element compliances from Table 2 and Equation (1) or the datasets and notebook provided through the links given in the data availability section.

**Table 2:** Element compliances $C_{i}^{-1}$ of KV-series fits to the average creep response at moisture content $mc$ values 0.07, 0.12, and 0.2, corresponding to 30, 65, and 90% relative humidity, respectively. For each case, the mean coefficient of determination $R^{2}$ represents the average of the $R^{2}$ values from the fits to the individual experiments

Loading direction	Transverse direction	Tissue type	*mc* [-]	$C_{i}^{-1}$	Mean $R^{2}$
*R*	*L*		0.07	[2.1,0.87,1.8,2.8]⋅10^−4^	0.92
			0.12	[4.2,12,9.6,7.6]⋅10^−4^	0.99
			0.2	[2.2,3.4,2.5,1.4]⋅10^−3^	1.00
*R*	*T*		0.07	[1.3,1.6,1.7,3.8]⋅10^−4^	0.95
			0.12	[3.0,14,9.8,4.8]⋅10^−4^	0.99
			0.2	[1.2,3.5,1.5,1.1]⋅10^−3^	0.99
*L*	*R*		0.07	[2.6,3.1,4.9,14]⋅10^−6^	0.90
			0.12	[3.9,4.4,9.6,19]⋅10^−6^	0.95
			0.2	[1.3,1.3,1.0,2.9]⋅10^−5^	0.95
*L*	*T*	*EW*	0.07	[5,7.3,9.4,23]⋅10^−6^	0.94
			0.12	[0.74,1.3,1.1,2.6]⋅10^−5^	0.97
			0.2	[2.8,2,2,3]⋅10^−5^	0.94
		*LW*	0.07	[1.8,2.9,3.1,5.8]⋅10^−6^	0.82
			0.12	[2.7,3.9,2.8,7.1]⋅10^−6^	0.95
			0.2	[6.5,6.4,7.6,13]⋅10^−6^	0.92
*T*	*R*		0.07	[6.9,3.9,9.6,18]⋅10^−4^	0.97
			0.12	[8.5,44,47,7.7]⋅10^−4^	0.99
			0.2	[4.3,14,3.7,2.6]⋅10^−3^	0.99

## Discussion

The cumulative overview of our findings, presented in Fig. 4 and Fig. 5, offers a comprehensive picture of the distinct impact of moisture and applied load on the creep behavior of spruce tissues loaded in different orientations. In general, the creep compliance $J_{c}$ increases with moisture and from L- to R- to T-direction, pointing at a strong directional dependence of the underlying deformation mechanisms. Notably, the impact of moisture varies significantly across the anatomical directions, following the same directional trend. However, the extent of its influence differs between creep and elastic compliance as their ratio $J_{c}/C_{0}^{-1}$ increases with moisture, revealing a more pronounced sensitivity of $J_{c}$.

From a micromechanical perspective, creep deformation involves a continuous adjustment of the cellular structure, which is qualitatively similar to elastic deformation but differs quantitatively due to the enduring application of a constant load. The outstanding creep performance in L-direction is explained by fiber stretching along their stiffest axis due to microfibril alignment. This is in contrast to cell wall bending in the transverse (R and T) directions, resulting in significantly higher compliance, even though to different extents (Gibson and Ashby 1997). Stretching deformation along the grain is also less sensitive to moisture and associated with one order of magnitude lower swelling than in transverse directions (Derome et al. 2011; Lanvermann et al. 2013; Persson 2000). As one should expect, EW exhibits higher $J_{c}$ than LW due to thinner cell walls, mainly due to a thinner S2 layer with higher MFA. Consequently, EW responds more readily to the applied load, promoting larger creep deformation as microfibrils have more potential to align with the load (El-Osta and Wellwood 1972; Roszyk et al. 2010, 2011). Therefore, when EW and LW are combined and loaded in parallel (LR samples), LW enhances the creep performance of the growth ring. Note that when EW and LW are loaded in parallel, they cannot be directly compared with the isolated tissues since, by sharing the same strain, they experience different loading degrees between each other and compared to the isolated tissues.

The described qualitative differences between elastic and creep deformation, in particular their interaction with moisture, must be interpreted across the different length scales, starting from the atomistic one. Creep activates a series of molecular mechanisms that differ from those involved in elastic deformation (Engelund and Salmén 2012). Elastic deformation is primarily driven by the lengthening and rotation of covalent and hydrogen bonds within the cell wall, while time-dependent deformation involves the breaking, movement, and reformation of hydrogen bonds between microfibrils and the embedding hemicellulose matrix (Amando De Barros and Wittel 2024). Since moisture primarily weakens hydrogen bonds, especially when water uptake is dominated by chemisorption (Niemz and Sonderegger 2017), its effect on creep is more pronounced than on elastic deformation, resulting in an increase in the $J_{c}/C_{0}^{-1}$ ratio with moisture. The energy required to stretch and break the bonds along the grain (Roszyk et al. 2011) is so high that $J_{c}/C_{0}^{-1}$ remains pretty low for all L samples up to 0.12 $mc$, and almost no difference can be seen between EW, LW, and their combination despite the differences in cell wall thickness and MFA. However, as relative humidity is high enough, so capillary condensation dominates, the energy barriers are lowered, accelerating creep compliance at high $mc$. A disproportionate acceleration of $J_{c}/C_{0}^{-1}$ is particularly evident in LW (Fig. 5c) and can be attributed to the distinct chemical composition and microfibrils-matrix interactions between EW and LW, especially within the dominant S2 layer. Since LW has a thicker S2 layer and higher hemicellulose content than EW (Bertaud and Holmbom 2004), it undergoes more pronounced swelling. Therefore, at high moisture levels, hemicellulose plays a key role in softening the cell wall material and weakening intralayer constraints, leading to a stronger reaction to moisture increase (Derome et al. 2011). Moreover, hemicellulose acts as a lubricant in microfibrils-matrix interactions, further enhancing the creep behavior of LW.

Any interpretation of transverse elastic or creep deformation must consider cellular phenomena, as observed deformations emerge from a complex interplay between cell wall bending and stretching in their softest directions. One observes that the T-direction is more affected by creep than R, as T-deformation is cell wall bending dominated, while R-deformation involves both bending and stretching, as the cell walls create slightly undulating load paths. One must expect that creep deformations of bent, layered cell walls are not homogeneous bending deformations but localized in cell corners where hinges form with high intra- and interlayer shear deformations. Besides this, one must also reflect on the crucial role of rays. In the R-direction, load is applied in the direction of the rays, causing them to continuously stretch and shrink, while in the T-direction, load acts perpendicular to the rays, which tend to open up, acting as microstructural voids. In the R-direction, a comparable trend was expected for *RT* and *RL*, given their shared loading direction. However, *RL* tends to exhibit greater compliance under high moisture. This counter-intuitive behavior is explainable by the sample preparation with microtome slicing, where *RL* samples expose open cells across the entire surface, while *RT* samples only reveal open cells along the edges. Additionally, rays in *RL* act as micromechanical voids due to their elongated shape along the length (L-direction), whereas in *RT*, rays are more evenly distributed (Ferrara and Wittel 2024). At high moisture levels, the softening of the cell walls amplifies the influence of these voids on $J_{c}$.

Both transverse directions show a pronounced increase with moisture in the chemisorption range (0.07 to 0.12 $mc$), where bending is the dominant underlying deformation mode. At higher moisture levels, $J_{c}$ continues to increase faster than $C_{0}^{-1}$, but tends to decelerate, suggesting a potential convergence to a constant ratio as fiber saturation is approached. The extreme softening of the cell walls at high moisture forces the fibers to deform, increasing the stretching component when cell walls align with the load direction for large deformations, even in the T-direction.

The investigation reveals a richness of phenomena that can only emerge through a comprehensive, comparative experimental campaign across different orientations, moisture levels, and LDs. However, creep testing is a time-intensive task. By classifying the tissue behavior into two primary clusters, namely the L-direction and the transverse directions, we can identify shared creep mechanisms that could potentially reduce the experimental effort. With some approximation, testing the L-direction and one transverse direction may suffice to estimate all uniaxial moisture-dependent components $J_{c,ii}$. Nonetheless, key differences between the R- and T-direction, such as the relative contributions of bending *vs* stretching and the role of rays, may require scaling factors to account for their unique behaviors within a unified framework.

## Conclusion

This extensive study provides valuable insights into the creep mechanisms of wood tissues across all anatomical directions under varying load and moisture conditions. Using a fully automated test rack with moisture control, multiple tissue samples were systematically tested, and the resulting creep compliance was characterized in terms of KV series-based models. The observed creep response results from a complex interplay between the intrinsic cellular properties of the wood and the geometry of its cellular structure, determining the deformation mechanism in each direction. The results highlight striking directional dependencies and reveal the moisture’s critical impact in modulating the different deformation mechanisms.

In the L-direction, EW showed greater creep compliance than LW due to its thinner walls and higher MFA. High energy is required to stretch and break matrix-cellulose bonds along the grain, but the transition from chemisorption to capillary condensation via physisorption accelerates creep compliance, particularly in LW with its thicker walls. In the transverse directions, the bending component produces a more compliant and moisture-sensitive creep response. However, cell wall stretching contributes to the elongation, especially at high deformations.

At the tissue level, natural variations in cellular structure are especially critical in thin samples, where features like rays significantly influence the overall response. Compared to macroscopic data, size effects in tissue samples are found to be more pronounced in creep tests than in elastic tests (Ferrara and Wittel 2024). At 150 h and 0.12 $mc$, the creep compliances in each anatomical direction are approximately twice as strong as those at the macroscopic scale in Maas and Wittel (2025), where the samples originate from the same tree as in this study. This distinction likely stems from the prolonged load application in creep tests, which activates time-dependent mechanisms sensitive to sample size. The size effect is intrinsic to the nature of the tissue samples. Their volume is large enough to be considered as representative for the different tissues, containing a significant number of fibers (approximately 1000 to 150000). However, they are not fully representative with respect to microstructural features such as rays. Moreover, the slicing process inevitably cuts open both cells and rays, which act as micromechanical voids. All these characteristics have a greater impact on tissue response due to the small thickness, while their influence is lower and more averaged in macroscopic samples. Increasing the sample thickness could help mitigate this effect and better homogenize the material behavior, but it would also shift the samples toward the macroscopic scale. The chosen thickness strikes a balance between technical feasibility and the ability to capture the mesoscale behavior of different tissues. This approach, for instance, allowed us to distinguish between RL and RT samples, despite their shared load direction. Therefore, the combined effects of cell wall mechanics and cellular geometry strongly shape the creep response of wood slices, often outweighing polymer interactions in the middle lamella, which mediate fiber-fiber sliding (Navi and Stanzl-Tschegg 2009).

All anatomical directions were explored in this study, and different tissue types were isolated for both LT and TL samples. However, only the results for LT samples allowed for a clear classification into EW and LW, as the data for TL samples proved unreliable. Future studies should address the limitations in TL sample preparation and measurement. Additionally, investigating the gradual transition of properties across a growth ring by using continuous slices, from EW to LW via transition wood, would further advance the understanding of the creep behavior across different anatomical directions and tissue types. It is known for wood that variations of properties between trees and even within one stem can be significant. To rule out those variations, we took all samples for all of our studies from adjacent regions of one tree. As a consequence, our results have reduced scatter and allow for a good comparison within the studies to reveal differences of mechanisms. A drawback, however, lies in the fact that numerical values are not of general character for spruce, since they are missing comparative values from different regions, trees, growth conditions, or even species. Long-term creep, beyond the three to seven days addressed in our work, could be the subject of further study as well. However, this will always be a compromise with data quality as it impacts the number of experiments per case.

## Figures and Tables

**Fig. A1:**
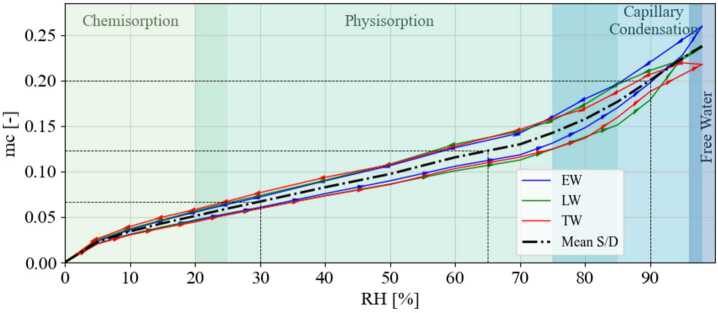
Experimental results of DVS tests on tissue samples of earlywood (EW), latewood (LW), and transition wood (TW). The mean curve (Mean S/D) represents the average of sorption (S) and desorption (D) curves, further averaged across tissue types

## Data Availability

The raw data can be obtained from 10.17632/vmvpvjj4g3.1, and the analysis tool from 10.5281/zenodo.14961188.

## Appendix A: Sorption isotherms

The dynamic vapor sorption (DVS) tests were conducted on tissue stripes using an automated sorption balance device (DVS Advantage ET85, Surface Measurement Systems Ltd.) following the procedure outlined in Koch et al. (2024). The results are provided in Fig. A1. Three different sorption/desorption (S/D) curves are shown, corresponding to different tissue types: EW, LW, and TW. Additionally, the mean water vapor isotherm is calculated as the average of the sorption and desorption curves, further averaged across tissue types. The mean S/D curve is plotted, highlighting the $mc$ values (0.07, 0.12, and 0.2) corresponding to the tested RH levels (30, 65, and 90%).

## Appendix B: Mechanical properties

**Table B1:** Mean values of linear elastic compliance $C_{0}^{-1}$ in MPa^−1^ and tensile strength $\sigma ^{f}_{ii}$ in MPa at three moisture content $mc$ values 0.07, 0.12, and 0.2, corresponding to 30, 65, and 90% relative humidity, respectively (Ferrara and Wittel 2024)

Mechanical property	Loading direction	Transverse direction	Tissue type	*mc* [-]	0.07	0.12	0.2
$C_{0}^{-1} \cdot 10^{-4}$ [MPa^−1^]	*R*	*L*			20 ± 2	23 ± 1	30 ± 3
	*R*	*T*			12.0 ± 0.5	15 ± 1	24 ± 2
	*L*	*R*			1.5 ± 0.1	1.6 ± 0.1	1.66 ± 0.07
	*L*	*T*	*EW*		2.0 ± 0.2	2.1 ± 0.3	2.7 ± 0.4
			*LW*		0.67 ± 0.05	0.73 ± 0.07	0.77 ± 0.08
	*T*	*R*			21 ± 4	34 ± 5	55 ± 5
$\sigma ^{f}_{ii}$ [MPa]	*R*	*L*			4.8 ± 0.4	4.8 ± 0.3	4.3 ± 0.3
	*R*	*T*			7.7 ± 0.6	7.6 ± 0.5	5.8 ± 0.3
	*L*	*R*			109 ± 5	108 ± 4	103 ± 6
	*L*	*T*	*EW*		67 ± 7	63 ± 6	61 ± 8
			*LW*		256 ± 24	239 ± 18	203 ± 26
	*T*	*R*			3.8 ± 0.5	3.2 ± 0.7	2.6 ± 0.4

The results of the mechanical properties obtained in our previous work (Ferrara and Wittel 2024) are reported here for consistency and reproducibility.

The mean tensile linear elastic compliance $C_{0}^{-1}$ along with the mean tensile strength $\sigma ^{f}_{ii}$ are summarized in Table B1. $C_{0}^{-1}$ is calculated as the inverse of the Young’s modulus ($E$), which is determined through linear regression applied to the strain range from about 10 to 60% of the ultimate strain. $\sigma ^{f}_{ii}$ is the maximum measured stress.
